# Safer opioid supply: qualitative program evaluation

**DOI:** 10.1186/s12954-023-00776-z

**Published:** 2023-04-20

**Authors:** Marlene Haines, Patrick O’Byrne

**Affiliations:** School of Nursing, University of Ottawa, Guindon Hall RGN 3051, 451 Smyth Road, Ottawa, ON K1H 8M5 Canada

**Keywords:** Safer Supply, Safer opioid supply, Overdose crisis, Harm reduction, People who use drugs, Qualitative research

## Abstract

**Background:**

As the overdose crisis in Canada continues to escalate in severity, novel interventions and programs are required. Safer Supply programs offer pharmaceutical-grade medication to people who use drugs to replace and decrease harms related to the toxic illicit drug supply. Given the paucity of research surrounding these programs, we sought to better understand the experience of being part of a Safer Supply program from the perspective of current participants.

**Methods:**

We completed semi-structured interviews and surveys with Safer Supply participants in Ottawa, Canada. Interviews were audio-recorded, transcribed, and analyzed thematically. Descriptive statistics were used to report survey data.

**Results:**

Participants most commonly discussed Safer Supply benefits. This included programs offering a sense of community, connection, hope for the future, and increased autonomy. Participants also described program concerns, such as restrictive protocols, inadequate drugs, and diversion.

**Conclusions:**

Our research demonstrated that participants found Safer Supply to be effective and impactful for their substance use goals. While participants did discuss concerns about the program, overall, we found that this is an important harm reduction-based program for people who use drugs in the midst of the overdose crisis.

## Introduction

The overdose crisis in Canada continues to escalate, with over 34,000 individuals having died from opioid toxicity between 2016 and 2023 [1]. These deaths have stemmed primarily from the toxic illicit drug supply, which initially became increasingly deadly in 2016 when fentanyl abruptly began to contaminate, then replace, heroin (diacetylmorphine) within the illicit drug market [2, 3]. More recently, the COVID-19 pandemic has exacerbated morbidity and mortality related to illicit opioid use, with opioid-related deaths increasing twofold–threefold, from 6 to 20 deaths per day from 2016 to 2023, respectively [1]. Additionally, new and concerning drugs are uncovered in the illicit drug supply weekly, with volatile additives such as metonitazene (a synthetic opioid), xylazine (a veterinary tranquilizer), and several benzodiazepine-related drugs [4]. The Canadian Institute for Health Information found that substance use disorders were the 4th most common reason Canadians were hospitalized between 2020 and 2021 [5].

Currently, opioid agonist treatment (OAT) is recognized as the gold standard treatment for individuals diagnosed with an opioid use disorder [6–8]. Typically, sublingual buprenorphine/naloxone is recommended as first-line therapy, followed by oral methadone [9, 10]. Other medication recommendations may include the use of naltrexone, injectable (subcutaneous) buprenorphine, and slow-release oral morphine (SROM) [6, 7, 11–13]. While the use of these medications is useful for many people who use drugs (PWUD), retention rates in care remain low. A recent study in Ontario, Canada, found that from 2014 to 2020, 58% of patients receiving OAT were not retained in treatment beyond 2 years—in fact, nearly 20% were retained for less than 90 days [14]. A randomized controlled trial in British Columbia, Canada, compared the use of injectable diacetylmorphine and oral methadone treatment for individuals who injected opioids daily. This study found that significantly more participants were retained in treatment with diacetylmorphine, compared to methadone (rate ratio for retention, 1.62; 95% confidence interval 1.35–1.95; *p* < 0.001) [15]. This demonstrated that while OAT is an essential and lifesaving treatment for many, there are portions of the population of PWUD who did not find OAT to be impactful or effective for their needs and goals.

To address the needs of PWUD who may not seek treatment for their opioid use or have not found OAT to be effective, Safer Opioid Supply programs have been implemented in a select number of communities across Canada. Safer Supply was first conceptualized by PWUD and has been formalized within a concept document written by the Canadian Association of People Who Use Drugs who define it as “a legal and regulated supply of drugs with mind/body altering properties that traditionally have been accessible only through the illicit drug market” [16]. Of importance, providing a Safer Supply of drugs is not limited to opioids. The concept of Safer Supply can be applied to any drug, including but not limited to stimulants, benzodiazepines, and hallucinogens. However, this particular research was undertaken with participants being prescribed opioids within a Safer Opioid Supply (which will be referred to as Safer Supply (SS) henceforth) program and thus, the focus will be on opioids.

It is important to note that while SS programs vary in the services they provide and their approaches to care, SS is not intended to be a treatment for opioid use disorder. Instead, it is a harm reduction-based concept which can be used to address the growing morbidity and mortality surrounding the toxic illicit drug supply. SS can be accessed in many different ways, ranging from “a Nurse Practitioner prescribing medications to an individual, to peer-led buyers’ clubs purchasing, testing, and distributing substances from the dark web” [17]. Outcomes related to SS programs may include—but are not limited to—impacting rates of overdose, participation in survival sex work, participation in criminalized behaviors, and changes in illicit substance use, as well as associated trauma and health concerns that may arise [18–20]. Most importantly, SS programs are designed and driven by client-centered goals and desires [21–25].

There is, however, a paucity of research evaluating SS programs, particularly from the perspective of SS participants. Thus, we completed a qualitative study with PWUD who were engaged with a SS program in Ottawa to better understand the experience of participating in a SS program. This included how the program impacts participants, as well as the facilitators and barriers to participating in a SS program.

## Methods

To effectively evaluate the SS program in Ottawa, semi-structured interviews and surveys were completed with program participants in the summer of 2022. To facilitate low-barrier access to participation, the interviewer went in person to each of the 3 SS program sites in Ottawa to conduct interviews. The first SS program exists within a supervised consumption site (SCS) attached to a homeless shelter in the Ottawa Downtown area. The SCS is open 24/7 while the SS program is open 16 h each day, from 0700 to 2300. This program is nurse-led and serves 46 participants. The second SS program is run within an addictions treatment clinic, open Monday–Friday for approximately 8 h each day. Physicians with additional addictions medicine meet with program participants and currently serve 361 participants in total. Finally, the third SS program exists within a community health center and is overseen by two nurse practitioners with training in addictions medicine. This program serves 69 participants. In total, the Ottawa SS programs serve 478 participants.

Overall, we had planned to recruit approximately 20–30 participants for in-depth, semi-structured interviews and surveys. This number is based upon qualitative research interview recommendations to ensure robust qualitative analysis [26, 27]. However, we ultimately used data saturation to decide when we had sufficient data collected from participants. Data saturation is described as the number of interviews required for “no new ideas to be mentioned [even] if more participants were sampled” [28 p. 1230], which typically occurs when 3 consecutive interviews do not yield new data.

Participants were recruited through posters advertising the study at each of the SS sites. SS program staff also informed participants about the study and when the interviewer would be on site. On the day of the interviews, participants were selected to participate on a first come, first served basis. Individuals were eligible to participate if they were 18 years of age or older, currently engaged in a SS program in Ottawa, and identify as a PWUD. There were no exclusion criteria.

### Data collection

At the outset of each interaction, an explanation of the project was provided to potential participants, including the purpose and objectives of the study. Participants reviewed and signed the consent form with the interviewer and were given $100 cash compensation for participating in the study.

Data collection was of two parts: (1) a 15–60-min audio-recorded semi-structured interview focused on discussing the experience of participating in a SS program, and (2) a 5–10-min survey to collect basic demographics and program outcomes. Semi-structured interviews were selected to allow for the stories and voices of SS participants to be prioritized, while still capturing important program information through a set of open-ended probes which were created in advance of data collection. See Table 1 for a list of interview probes.

**Table 1:** Interview probes

Topic	Questions
Program intake	When did you complete the SS intake process? Why did you want to start the program? How did you find the intake process? What did you like about the intake process? What did you not like about the intake process?
Program check-ins	How often do you complete checks ins with your SS team? What do you like about the check-ins? What do you not like about the check-ins? Is there anything you want to change/add/take away from your check-ins?
Health impacts	Has your health changed at all since starting a SS program? Physical health? Mental health? Were you experiencing overdoses prior to starting SS? If yes, has the amount/frequency changed since starting the program?
Social impacts	How do you feel about being part of a SS program? How do you feel you are treated as a participant in a SS program—for example, how do you feel you are treated by: SS nurses? SS nurses? Other SS staff? Peers? Family? Friends? Other PWUD? Hospital staff? Prison staff?
Substance use	Has your substance use changed since starting SS? If yes, please describe
Criminal activity	Were you participating in any criminal activity prior to starting SS to access illicit substances? If yes, has this changed at all since starting the program?
Goals	What were your goals when starting SS? Were you able to meet any of your goals? Did anyone help you meet your goals?
Program setup	How do you feel about the environment you access your SS in? How do you feel about the hours of operation? How do you feel about the current staffing models? What might you like to see changed?
Resources and expansion	What would you want policy makers to know about SS? What research would you want to be done to investigate SS further? What supports do you wish you could access alongside SS? What supports do you wish existed for yourself? What supports do you wish existed for your peers?
Concerns	Do you have any concerns about your SS program? Do you feel that there are issues surrounding diversion of SS medication? Why or why not?

### Data analysis

Following the completion of the interviews, all audio recordings were transcribed by a professional transcription service. Qualitative data analysis occurred as per Smith, Flowers, and Larkin [29]. Both authors separately read through each transcription multiple times, forming initial notes and codes regarding what the participants discussed. The authors then compared their notes and codes to ensure congruency in initial findings and discuss any inconsistencies. Notes and codes were then clustered together to form larger themes to allow for the essence of the experience to be revealed. Descriptive statistics were used to report the data collected from the self-administered surveys. Of note, there was consistency within the themes brought forward by participants at all SS sites regarding their program experiences. Thus, participant results were reported together.

## Results

### Surveys

A total of 30 participants completed an interview and survey (see Tables 2, 3, 4, 5, 6 and 7). Participants from all 3 existing SS programs in Ottawa were recruited, including 14 from a clinic, 12 from a shelter/SCS, and 4 from a community health center. In addition to being part of a SS program, 8 participants were also part of a Safer Stimulant Supply program. All participants report being prescribed some form of a long-acting opioid medication as part of their SS program—the most common being SROM only for nearly half of the participants. Participants had been part of the SS program for a median length of 20.5 months (IQR 15–30). The majority of participants self-identified as male (57%) and the median age was 42 years old (IQR 35–50). Just over half of the participants (53%) identified as Caucasian, while 20% identified as Indigenous.

**Table 2:** Baseline demographic characteristics of the program participants

Characteristics	Participants (*n* = 30)
Age (years)	42 (35–50)
Time on safer opioid supply program (months)	20.5 (15–30)
Self-identified gender	
Female	13 (43.3%)
Male	17 (56.7%)
Ethnicity	
Caucasian	16 (53.3%)
Indigenous	9 (30%)
Mixed ethnicity	4 (13.3%)
Other	1 (3.3%)
Sexual orientation	
Heterosexual	27 (90%)
Bisexual	2 (6.7%)
I don’t know	1 (3.3%)
Country of birth	
Canada	28 (93.3%)
Other	2 (6.7%)
Spoken languages	
English only	15 (50%)
English and French	10 (33.3%)
Multilingual	5 (16.7%)
Highest level of education	
Less than high school	16 (53.3%)
High school	4 (13.3%)
Trade school	1 (3.3%)
Some college	6 (20%)
College	1 (3.3%)
Some university	2 (6.7%)
Current long-acting opioid	
SROM (24-h)	14 (46.7%)
Buprenorphone/Naloxone	1 (3.3%)
Methadone	3 (10%)
Methadone and SROM (24-h)	9 (30%)
Methadone and SROM (12-h)	3 (10%)
Program site	
Clinic	14 (46.7%)
Shelter/SCS	12 (40%)
Community health center	4 (13.3%)
Safer stimulants	
Yes	8 (26.7%)
No	22 (73.3%)

Data are expressed as median (IQR) for continuous variables and number of participants (%) for categorical variables

**Table 3:** Substance use among participants

	Participants (*n* = 30)
Age when first used drugs	13 (11–15)
Age when first used opioids	22 (18–33)
Lifetime drug use	
Cocaine	30 (100%)
Crack cocaine	29 (96.7%)
Crystal methamphetamine	26 (86.7%)
Diacetylmorphine (Heroin)	28 (93.3%)
Fentanyl	29 (96.7%)
Other opioids	29 (96.7%)
Benzodiazepines	28 (93.3%)
Alcohol	26 (86.7%)
Cannabis	28 (93.3%)
Prescription stimulants	22 (73.3%)
Inhalants	11 (36.7%)
Hallucinogens	26 (86.7%)
Anabolic steroids	5 (16.7%)

Data are expressed as median (IQR) for continuous variables and number of participants (%) for categorical variables

**Table 4:** Illicit substance use complications prior to Safer Opioid Supply program

Complication	Participants (*n* = 30)
Abscesses or skin infections	21 (70%)
Frequent overdoses	26 (86.7%)
Hospital visits	26 (86.7%)
Endocarditis	1 (3.3%)
HIV	2 (6.7%)
Hepatitis C	26 (86.7%)
Legal issues	29 (96.7%)

**Table 5:** Drug use locations

Location	Participants (*n* = 30)
Supervised consumption site	29 (96.7%)
Home/someone else’s home	29 (96.7%)
Public/outside	27 (90%)
Shelter	19 (63.3%)

**Table 6:** Harm reduction service access

Service	Participants (*n* = 30)
*Supervised consumption sites*	
Ottawa Inner City Health	26 (86.7%)
Ottawa Public Health	18 (60%)
Sandy Hill CHC	25 (83.3%)
Somerset West CHC	23 (76.7%)
*Harm reduction services*	
Sterile site distribution	29 (96.7%)
Needle exchange	30 (100%)
Take home Naloxone	29 (96.7%)
Condoms/contraceptives	15 (50%)
Peer services/outreach	26 (86.7%)
STBBI testing	29 (96.7%)
Education^a^	22 (73.3%)
Infectious disease services^b^	28 (93.3%)

^a^Education on sex work, infection prevention, overdose prevention, etc.

^b^Infectious disease testing/prevention (e.g., TB, STI, HIV, vaccines, Hep A/B/C, etc.)

**Table 7:** Before/after Safer Opioid Supply

Measure	Before	After
	*n* = 30^a^	*n* = 30^a^
Fentanyl use (points/day)^a^	10 (5–15)	1.5 (0.5–4)
Mental health ratings^b^	1.75 (1–3)	3.75 (3–4)
Overdose events		
Any overdose	28 (93.3%)	6 (20%)
No overdose	2 (6.7%)	24 (80%)
Legal issues		
Any legal issues	28 (93.3%)	12 (40%)
No legal issues	2 (6.7%)	18 (60%)
Housing status		
Housed	8 (26.7%)	15 (50%)
Unstably housed	0	1 (3.3%)
Shelter	22 (73.3%)	14 (46.6%)
Income source		
Ontario works	15 (50%)	7 (23.3%)
ODSP	12 (40%)	23 (76.7%)
Employment	1 (3.3%)	0
None	2 (6.7%)	0

Data are expressed as median (IQR) for continuous variables and number of participants (%) for categorical variables

^a^Only includes participants reporting fentanyl use (*n* = 26)

^b^Participants were asked to rate their mental health on a scale of 0–5, with 0 being very poor and 5 being excellent

All participants reported complications as a result of their illicit drug use prior to beginning SS. The most common complications reported were legal issues (97%), frequent overdoses (87%), hospital visits (87%), and Hepatitis C infections (87%). The most common locations participants reported using drugs included at a supervised consumption site (97%) and at home/someone else’s home (97%). All participants regularly access harm reduction services, including needle exchange (100%), sterile site distribution (97%), and sexually transmitted and blood-borne infection testing (97%).

### Interviews

Within the semi-structured interviews, participants were asked to discuss their experiences within their SS programs. Participants highlighted SS program concerns and benefits.

#### Program concerns

##### Prescribing standards

Although participants most often wanted to speak about the benefits of SS programs, there were a few concerns raised. First, several participants noted that current SS prescribing standards in Ottawa (Hydromorphone 8 mg × 30 tabs/day = Hydromorphone 240 mg/day maximum) were inadequate to combat the ensuing withdrawals and cravings associated with illicit fentanyl. One participant noted that early on in their program their Hydromorphone dose rapidly “became just not enough. I got the tolerance to that as well… [it made me feel] worthless. Sad” (P21). Participants nevertheless remained hopeful, stating that, “the Dilaudid is still a little short, but it's getting there” (P4), and that, while the current Hydromorphone dosing “doesn’t do what they used to do, because they're way lighter than the fentanyl, they still help” (P9). Along with concerns regarding maximum dosages, one participant complained of feeling like current dose increases were inadequate for their needs: “the start was a little low… and then if I missed a day, I got shot back down” (P7).

##### Restrictive protocols and policies

Several participants found different aspects of the SS program to be restrictive. Some individuals began their program by having their doses witnessed—a staff member would monitor them as they prepared and injected the Hydromorphone tablets or took them orally. Within this model, the number of tablets SS participants could access at one time was restricted (e.g., participants could access Hydromorphone 8 mg × 1–6 tabs every 1–2 h). One participant stated, “I'd like to have them all because I want to do them any time I want” (P3). Another reflected on the lack of control they had when they started the program:

At first, it [Safer Supply] was not as good because I had to do supervised doses, but after that it was okay… I couldn’t do as much I wanted. When I'm at home I can use a 3 mL barrel and I can put more pills in it. It's a bit of more of a process, but it’s like doing it at home better. (P2)

Certain SS programs placed more regimented requirements on medication pick-ups and check-ins with clients. One client found picking up their medication at the pharmacy each day to be onerous, noting it can be “a little annoying especially when they go all year round” (P28). Another participant cited a time they asked their prescriber to alter their pickup schedule:

I said, “Well, can I pick them up every other day?” And he said, “No.” But that would be a good thing, every other day. If you could give me my dosage for two days, that would be fantastic. (P7)

Our participants reported similar sentiments regarding weekly check-ins by participants whose program mandated this. Participants also found the lack of portability within SS programs to be frustrating—one participant recounted a desire to see his family in another province: “it would be nice to even get up and out of town just for a bit and do my Safer Supply still” (P26).

##### Medication diversion

Finally, a number of participants commented on medication diversion [30]. Most participants acknowledged that diversion of medication did occur at times, with one participant recounting a time they were asked to divert: “In the beginning, people were asking me and I told them it’s not happening. I’m not messing up my program” (P17). Several participants noted that they did not agree with others who were diverting medication: “I think they’re crazy, myself” (P15). However, many participants were also able to provide important insights into why diversion happened. Given the vast difference in potency between Hydromorphone and illicit fentanyl, participants pointed out that:

People trade their Dilaudid because they want the fentanyl that is strong enough to overpower the fentanyl that they were using before the Dilaudid program. The Dilaudid program only offers 30 pills maximum, which is nowhere near as high an amount as the fentanyl is. (P28)

Participants also explored the fact that “if somebody’s coming down here and trying to look for fentanyl, but they can only find Dilaudid, it’s going to be a lot safer for them” (P11). Another participant noted that if “they’re willing to… buy some more [Hydromorphone] to avoid the usage of other things, then by all means, sure” (P13). The cultural expectation of supporting and sharing with other PWUD was underscored: “it’s just a couple pills, right?” (P19). Some participants felt it was unacceptable for them to withhold medication that could help someone else: “I'll give one or two away if somebody’s hurting. Of course, I will. And I hold no shame in that” (P27). Before SS, participants relied on their community when they had no opioids to use—with or without a SS program, reciprocity is an expectation and demonstration of care:

If people are both on the same medications, then sharing shouldn’t be a problem. If you’re both on 8 Dilaudids and I run out or I lose mine today or somebody fucking jacks me for them or… [if] I fall asleep and somebody steals them all, I don’t see a problem with somebody helping somebody out for the day… I don’t see anything wrong with it. (P24)

Of importance, no participants reported knowledge of any selling or sharing that occurred with people who were not known to use opioids. Selling and sharing opioids—whether related to SS or not—only occurred between and within PWUD who knew each other.

#### Program benefits

##### Consistency

Participants spoke at length about the different ways that SS had benefitted their lives. The knowledge of understanding exactly what was in the medication they were using was powerful—several participants contrasted this knowledge with their previous experiences using the toxic illicit drug supply: “At least with [Safer Supply medication], I know what I’m getting and I don't have to worry like ‘today, I’m going to go [overdose]’” (P8). Individuals felt confident in the medication provided to them, with one participating stating, *“*because I just know it's pharmaceutical class… there’s no stress involved” (P14) and another noting increased levels of control, “I know by milligram or whatever how much I’m using. So, I can almost like plan it in a day, how much [Safer Supply medication] I’m going to use every time” (P1).

##### Safety

Participants vocalized the sense of safety that SS provided them with: “once I was a client of this program, I knew I was safe” (P28). There was a sense of relief associated with no longer having to participate in criminalized behaviors to avoid being dope sick: “the safety net thing… not having to worry about breaking the law to get [opioids], or just having every day taken care of to get stabilized” (P2). Importantly, simply having the knowledge of when and where SS can be accessed next alleviated anxiety and caused withdrawal symptoms to be less worrisome for a participant:

The first and foremost thing about Safe Supply is the comfort in knowing that it’s only going to be a little bit longer before I can get better, you know? So, when the sickness comes, whether it’s at 8 o’clock at night and I start to feel withdrawal, I can tell myself, I just have to wait until 8 in the morning and then things are going to be OK. Things are going to get better. And that has helped 1000%. Just the knowledge that it’s going to be there and I don’t have to worry about it. (P10)

##### Community

While our participants recounted how access to consistent medication was essential, they also spoke at length about the sense of community their SS program provided to them. Two different participants described the SS staff as their family, while another similarly said, “I feel like I’m taken care of. I feel like I’m looked after. I feel like I’m cared about, I feel like I have a whole other family” (P1). A deep sense of community was clearly rooted in the SS programs, making participants feel accepted and welcomed. This resulted in participants feeling listened to and cared for: “when they ask how you're doing, they really want to know how you’re doing” (P22).

##### Trust and respect

Along with this sense of community, participants felt extremely connected to the staff they worked with on such a consistent basis. Participants described this feeling as “like you’re walking into friends [rather] than going to see a doctor, I find that helps a lot” (P8). While participants noted being engaged in the process of the SS program themselves, they also described feeling as though the staff were engaged with and invested in their success as well. Further, when peers/people with lived experience were present in SS programs, this heightened connection and bonding:

Some of these people, you never, ever, ever think that some of these people have had the past that you had. And it gives you inspiration. Like once they open up, and they tell you, so like, “Hey, this is what I've been through.” It’s a real eye-opener. If they can do it, why can’t I?” (P11)

SS staff provided a program where participants felt they could regain some semblance of trust and respect within the healthcare system. Participants spoke about previous instances of being treated poorly by nurses or doctors in other settings and spoke about the fact that “I don’t have a lot of people that I can trust enough to talk to and stuff, and I trust her completely. I can tell them anything about my use… everything (P29). Although developing trust took time, participants found that staff allowed them to “feel like a human being… there’s nothing to hide here” (P18).

##### Gratitude

Participants also commonly expressed gratitude for participating in the program. SS programs were not available in most communities, and even in places with programs, spaces were often limited. One participant noted, “It’s saved my life because I would've died probably on fentanyl” (P17). Other participants echoed this, viewing the opportunity as a turning point in their life:

I love being in it. I’m grateful I got in it. I didn’t think I was, and then they just recently shut down accepting patients, so I feel grateful that I’d got in. I feel like it gave me a chance to save my life. (P23)

Many participants reflected on peers and friends who did not have a chance to try SS, and who had died due to illicit fentanyl use: “I’m lucky to be here because a lot of my friends have passed and are dead now. So, I feel grateful” (P18). With this, participants described feeling more optimistic and generally having “more hope in my life” (P14). Prior to starting SS, many participants described feeling as though they had no future and could die at any moment. In contrast, when asked if they feel differently about the future since starting SS, one participant stated: “I don’t know about a different future. I just know that there is a future. So, that’s a start right there” (P18).

##### Autonomy

Participants often disclosed feeling as though they had little decision-making power surrounding their substance use prior to SS. They described having no choice but to access the dangerous and toxic illicit opioid supply day to day to manage their ongoing withdrawal symptoms and cravings. When they did engage in accessing supports (e.g., OAT, detox, rehabilitation programs) they were forced to adhere to specific and pre-determined policies and protocols. Joining SS meant “not feeling like we were in handcuffs anymore” (P9). Participants enjoyed the fact that they had the freedom to “be in control of how we use it and dose” (P14) their SS medication. This autonomy meant that they could “just come here and get my pills and go about my day” (P24).

##### Support and stability

In contrast to discussions surrounding autonomy, participants also described the support and stability provided by SS programs as essential. One participant described this as “the stability of not having to run around and get that money and get that stuff [fentanyl] every day” (P24). Another stated that SS provided “more support for opportunities to let go of the street drugs” (P4) which resulted in “finally getting to a point where we could think of other things than just doing drugs” (P1).

##### Structure

The need to pick up their medication each day and check in with their team at regular intervals allowed many participants to develop a structured schedule. Participants recounted settling into this new routine and how “it’s easier to do learned behavior multiple times” (P6). Another participant described how SS without the structure and support would not have worked for them:

I got myself into a routine. Coming in every day, seeing staff… it was the whole thing… It just puts you in a whole other mindset… I think if it was just me coming and going into a pharmacy and picking up the Dilaudids, and doing them [Safer Supply] that way, I don’t think it would work. (P11)

Another participant acknowledged that at times this routine could be restrictive, but that in the end it was necessary for their success: “it’s a pain in the ass, going back and forth, but for me it’s important, because I need that daily contact… they [Safer Supply staff] know me. They make people feel like people” (P22).

##### Pre-/post- program measures

In the interviews, participants were asked to elaborate on the responses they provided for pre-post SS program measures. Many participants spoke about how since starting SS they “don’t do any crime whatsoever” (P25) and that SS “gave me a chance to stay out of jail. I’ve been 2 years now without any police problems… it’s never usually like that for me” (P2). Several participants described an intense feeling of relief associated with this change, with one participant describing that they “never think about it [crime]. It makes me happy that I’m free… looking over my shoulder all the time, I don’t have that feeling anymore” (P7). Importantly, even among those who continued to participate in criminalized behavior out of necessity, the vast majority report that they are “doing a lot less” (P4) and the overall, SS allows them to “try to stay away from doing crime” (P30).

With regards to substance use, many participants who were using illicit fentanyl prior to the program reported that they “no longer use fentanyl” (P14), “I’m off fentanyl” (P17). While some participants did continue to use fentanyl, all reported that their “need to use more fentanyl has decreased greatly” (P13). One participant articulated this change as “like night and day…I was using at least a gram a day [of fentanyl], and I'm now using maybe a point a day” (P5). In addition to decreasing their illicit opioid use, participants described other drug-related benefits, such as “not smoking nearly as much crack as I was” (P23) and the fact that they “don’t inject as much” (P28). Several participants also noted that they “don't overdose anymore” (P18), while others found their rate of overdose to be notably decreased:

I would usually be overdosing at least a few times a week or at least a couple. Now it’s been one overdose in a month or two, so it’s definitely a big improvement. It [Safer Supply] helps with the cravings and helps me try and stay out of the cycle of repetitively draining my energy or my willpower. (P12)

Lastly, all participants noted how their overall health and wellness benefitted over their time in SS. Activities of daily living such as showering, eating, and getting out of bed were more attainable goals: “I believe I function much better with opioids in my system. I keep myself clean, pay the bills, have a relationship with my child, everything, have a normal life” (P15). Several participants noted improvements in their physical health, such as healthy weight gain (“I’ve gained about 50 pounds” P27), improved stamina (“I’m able to get around more…because I'm not in as much pain all the time” P29), and better self-care (“I rest when I need to rest” P10) since starting SS. One participant noted the SS allowed her to re-engage in the healthcare system:

I did what I had to, go to the hospital, get my surgery. And those are things I wouldn’t have done if I wouldn’t have been on Safer Supply… Because I didn’t care as much. Being on Safer Supply helped me take care of my health. (P5)

Improvements to mental health were also discussed, such as decreased stress, “learning to cope with things better” (P6), and improved memory (“the longer you don’t use [fentanyl] and overdose, the better your memory seems to become” P18). One participant spoke at length about their previous experience of being in and out of psychiatric institutions on a near-weekly basis. They noted that once they started to use opioids, their cycling through mental health services stopped:

I've been in and out of psych ward my whole life because of that [my trauma]. And I haven’t been in a psych ward for probably 15 years now… that's as long as I’ve been doing opioids. (P25)

For this participant, opioids were the medication they needed to manage their complex trauma. However, their previous attempts with abstinence or treatment-based care for their substance use care were not effective. Instead, SS allowed them to continue managing their mental health concerns with their medication preference of short-acting opioids.

## Discussion

In this paper, we reported on the findings from interviews and surveys completed with 30 persons in Ottawa, Canada about their experiences participating in a SS program. Overall, we found that participants reported improvements to their safety and well-being since joining SS, such as improved mental health, reductions in criminalized behavior, decreased overdose events, and reduced illicit drug use. Participants overwhelmingly wished to speak about the numerous benefits they derived from being part of a SS program, such as a sense of community and connection, hope for the future, safety in their drug use, and autonomy. However, participants did discuss concerns with the program, such as inadequate drugs, diversion, and restrictions. These findings raise a couple of points for discussion.

Firstly, participants spoke at length about the multitude of benefits they derived from having access to a SS program. This contrasted starkly with participants describing the toxic illicit drug supply before SS as dangerous. Accessing medication at regular intervals also provided participants with comfort, as it enabled them to know when and how they would obtain their next dose of medication to manage opioid cravings and withdrawals and mitigate the need for participation in criminalized behavior to acquire the resources to obtain illicit drugs. These findings echo previous research which found that SS participants reported how having a reliable source of drugs assisted with decreasing their risk of overdose, minimizing their need to participate in criminalized behaviors, and provided them more control over their drug use [20]. Of note, the community of PWUD attempting to mitigate risk through accessing safer drugs is not novel to SS programs. Several studies have demonstrated how PWUD rely on drug dealers they have developed trust with to safely navigate the illicit drug supply [31, 32], and the harm reduction methods (e.g., drug checking) many dealers report participating in to provide a SS from the illicit drug markets [33].

Secondly, the findings of community, care, connection, and trust were emphasized by all research participants. While the main objective of these SS programs was to offer prescription medication to participants, they also incorporated wraparound services such as housing workers, peer supports, and primary care connections into SS programs. Further, the participants reported their SS staff helped to create an environment of safety and trust. This is in contrast to the stigma and shame PWUD commonly report when accessing healthcare services [34–36]. However, it is consistent with findings from other harm reduction service research, such as participants at an overdose prevention site in Toronto emphasizing the program providing a sense of belonging and community [37].

Thirdly, when discussing program concerns, there was a contrast between participants who found SS protocols to be restrictive and those who enjoyed the structure and routine they developed. These opposing views highlight the need for SS and other substance use programs to approach care plans with flexibility and adaptability to support participant success. Previous research with PWUD has demonstrated that rigid substance use program protocols and policies (e.g., lengthy medication titration timelines, low starting doses, missed dose protocols, etc.) can act as a barrier to care [38, 39]. Further, this underscores the need to include PWUD in the conceptualization, design, implementation, and evaluation of programs and services with a focus on substance use [40].

Fourthly, the need for SS medication to align with illicit drug use remains a potential gap in care. While fentanyl and fentanyl analogs dominate the illicit drug market, the majority of SS programs predominantly offer hydromorphone [41]. Research has shown that PWUD describe fentanyl as extremely potent with a rapid onset when compared to other opioids [2]. A recent study found that the majority of PWUD preferred heroin (57.8%), followed by fentanyl (32.8%), and finally prescription opioids (9.4%) [42]. This differs from our participants, who articulated a preference for injectable fentanyl in their interviews, and most did not mention heroin. This contrast may be due to the aforementioned study occurring in 2019; although fentanyl was present at this time, it was not as prolific in the toxic illicit drug supply as it is currently. Nonetheless, improved access to different opioid formulations (e.g., injectable fentanyl, diacetylmorphine) is an important point that warrants further clinical and research consideration.

Lastly, despite diversion being a commonly discussed issue concerning SS programs [30], most participants did not feel diversion was a central issue. They were also clear that this diversion only involved persons already using drugs and did not occur involving persons who did not already use these substances. Sharing drugs and keeping peers safe is a normal and expected part of the community of PWUD—diversion is furthermore not unique to SS medication [30, 43, 44]. Further, when PWUD were faced with a toxic illicit drug supply, electing to share or purchase pharmaceutical-grade medication can be seen as a rational, protective harm reduction measure to avoid overdose death [45]. A document recently released by the SS National Community of Practice helps to reframe diversion by dividing it into unique categories, such as compassionate or survival sharing [30]. Further, other harm reduction programs such as SCS are moving toward allowing splitting and sharing of drugs to align with current rules of engagement among PWUD [43].

### Limitations

It is important to note the limitations of this research. Survey responses were collected by self-report, increasing the possibility that participants provided inaccurate data based on what they believed the research team wanted to hear. To mitigate this risk, participants were informed that the research data collected would be kept confidential and have no bearing over or relation to their SS program. This research also did not include a control group, meaning it can be difficult to draw objective conclusions about the impacts of SS programs. However, given this study was primarily initiated to understand the personal experience of participating in a SS program, a control group was not necessary.

## Conclusion

With the overdose crisis escalating in severity, novel solutions and programs are required to suit the different needs of PWUD. SS program providers in Ottawa have sought to offer a harm reduction-based option for PWUD to access substances consistently and safely. While participants did discuss some concerns within the programs, such as restrictions, inadequate drugs, and diversion, the vast majority of participants emphasized the benefits of these programs throughout their interviews. Participants appreciated being part of a SS community, often developing connection and trust with care providers. They felt immense gratitude and hope for the future demonstrated through the consistency of care they felt they were receiving. Finally, SS programs offered increased autonomy, while simultaneously providing structure and stability within their lives.

## Data Availability

The datasets used and/or analyzed during the current study are available from the corresponding author upon reasonable request.

## Abbreviations

OAT: Opioid agonist treatment
PWUD: People who use drugs
SROM: Slow-release oral morphine
SS: Safer Supply

## References

[1] Special Advisory Committee on the Epidemic of Opioid Overdoses. Opioid- and stimulant-related harms in Canada, https://health-infobase.canada.ca/substance-related-harms/opioids-stimulants/. 2023.

[2] Mayer S, Boyd J, Collins A (2018). Characterizing fentanyl-related overdoses and implications for overdose response: findings from a rapid ethnographic study in Vancouver, Canada. Drug Alcohol Depend.

[3] Hayashi K, Milloy MJ, Lysyshyn M (2018). Substance use patterns associated with recent exposure to fentanyl among people who inject drugs in Vancouver, Canada: a cross-sectional urine toxicology screening study. Drug Alcohol Depend.

[4] Centre on Drug Policy Evaluation. What’s in Toronto Drug Supply? 2022. https://drugchecking.cdpe.org. Accessed 6 July 2022.

[5] Canadian Institute for Health Information. Hospitals stays in Canada. 2022. https://www.cihi.ca/en/hospital-stays-in-canada. Accessed 6 July 2022.

[6] British Columbia Centre on Substance Use. A guideline for the clinical management of opioid use disorder, http://www.bccsu.ca/care-guidance-publications/. June 2017.

[7] Canadian Research Initiative in Substance Misuse. CRISM National guideline for the clinical management of opioid use disorder. 2018. https://crism.ca/projects/opioid-guideline/. Accessed 1 Aug 2022.

[8] Centre for Addiction and Mental Health. Opioid agonist therapy: a synthesis of Canadian guidelines for treating opioid use disorder. 2021. https://www.camh.ca/-/media/files/professionals/canadian-opioid-use-disorder-guideline2021-pdf.pdf. Accessed 1 Aug 2022.

[9] Handford C. Buprenorphine/naloxone for opioid dependence: clinical practice guideline. 2012. https://www.porticonetwork.ca/documents/489955/0/Buprenorphine+and+Naloxone+-+Clinical+Practice+Guidelines+2012+PDF/fd1eee5d-fd7f-4b58-961f-b45350a8b554. Accessed 1 Aug 2022.

[10] Bromley L, Kahan M, Regenstreif L, et al. Methadone treatment for people who use fentanyl: Recommendations. Toronto. 2021. https://www.metaphi.ca/wp-content/uploads/Guide_MethadoneForFentanyl.pdf. Accessed 1 Aug 2022.

[11] Équipe de soutien clinique et organisationnel en dépendance et itinérance. Guide to using slow-release oral morphine (Kadian^®^) in opioid agonist therapy (OAT). 2021. http://dependanceitinerance.ca/wp-content/uploads/2021/06/GRAPHISME-Outil-Kadian-EN-210622.pdf. Accessed 1 Aug 2022.

[12] Nasser AF, Greenwald MK, Vince B (2016). Sustained-release buprenorphine (RBP-6000) blocks the effects of opioid challenge with hydromorphone in subjects with opioid use disorder. J Clin Psychopharmacol.

[13] Haight BR, Learned SM, Laffont CM (2019). Efficacy and safety of a monthly buprenorphine depot injection for opioid use disorder: a multicentre, randomised, double-blind, placebo-controlled, phase 3 trial. The Lancet.

[14] Tahsin F, Morin KA, Vojtesek F (2022). Measuring treatment attrition at various stages of engagement in opioid agonist treatment in Ontario Canada using a cascade of care framework. BMC Health Serv Res.

[15] Oviedo-Joekes E, Brissette S, Marsh DC (2009). Diacetylmorphine versus methadone for the treatment of opioid addiction. N Engl J Med.

[16] Canadian Association of People Who Use Drugs. Safe supply: concept document. Dartmouth, https://www.capud.ca/capud-resources/safe-supply-projects. 2019.

[17] Haines M, Tefoglou A, O’Byrne P. Safer supply Ottawa evaluation: fall 2022 report. Ottawa, https://safersupplyottawa.com/research/. 2022.

[18] Gomes T, Kolla G, McCormack D (2022). Clinical outcomes and health care costs among people entering a safer opioid supply program in Ontario. Can Med Assoc J.

[19] Ivsins A, Boyd J, Mayer S (2020). ‘It’s helped me a lot, just like to stay alive’: a qualitative analysis of outcomes of a novel hydromorphone tablet distribution program in Vancouver, Canada. J Urban Health.

[20] Ivsins A, Boyd J, Mayer S (2020). Barriers and facilitators to a novel low-barrier hydromorphone distribution program in Vancouver, Canada: a qualitative study. Drug Alcohol Depend.

[21] Ranger C, Hobbs H, Cameron F, et al. A practice brief: implementing the Victoria SAFER initiative. Vancouver, https://www.uvic.ca/research/centres/cisur/assets/docs/colab/practice-brief-safer.pdf (2021).

[22] Tyndall M (2020). A safer drug supply: a pragmatic and ethical response to the overdose crisis. Can Med Assoc J.

[23] Young S, Kolla G, McCormack D (2022). Characterizing safer supply prescribing of immediate release hydromorphone for individuals with opioid use disorder across Ontario, Canada. Int J Drug Policy.

[24] Glegg S, McCrae K, Kolla G (2022). ‘COVID just kind of opened a can of whoop-ass’: the rapid growth of safer supply prescribing during the pandemic documented through an environmental scan of addiction and harm reduction services in Canada. Int J Drug Policy.

[25] Ivsins A, Boyd J, Beletsky L (2020). Tackling the overdose crisis: the role of safe supply. Int J Drug Policy.

[26] Dworkin SL (2012). Sample size policy for qualitative studies using in-depth interviews. Arch Sex Behav.

[27] Adams WC. Conducting semi-structured interviews. In: Newcomer KE, Hatry HP, Wholey JS (eds) Handbook of practical program evaluation. Hoboken, 2015, pp. 492–505.

[28] Francis JJ, Johnston M, Robertson C (2010). What is an adequate sample size? Operationalising data saturation for theory-based interview studies. Psychol Health.

[29] Smith JA, Flowers P, Larkin M (2009). Interpretative phenomenological analysis: theory, method, and research.

[30] National Safer Supply Community of Practice. Reframing diversion for health care providers: frequently asked questions, https://www.nss-aps.ca/reframing-diversion-prescribers (2022).

[31] Carroll JJ, Rich JD, Green TC (2020). The protective effect of trusted dealers against opioid overdose in the U.S.. Int J Drug Policy.

[32] Bardwell G, Boyd J, Arredondo J (2019). Trusting the source: the potential role of drug dealers in reducing drug-related harms via drug checking. Drug Alcohol Depend.

[33] Betsos A, Valleriani J, Boyd J (2021). I couldn’t live with killing one of my friends or anybody: a rapid ethnographic study of drug sellers’ use of drug checking. Int J Drug Policy.

[34] Muncan B, Walters SM, Ezell J (2020). ‘They look at us like junkies’: Influences of drug use stigma on the healthcare engagement of people who inject drugs in New York City. Harm Reduct J.

[35] Biancarelli DL, Biello KB, Childs E (2019). Strategies used by people who inject drugs to avoid stigma in healthcare settings. Drug Alcohol Depend.

[36] Brezing C, Marcovitz D, Parekh R, Childs EW (2016). Stigma and persons with substance use disorders. Stigma and prejudice.

[37] Foreman-Mackey A, Bayoumi AM, Miskovic M (2019). ‘It’s our safe sanctuary’: experiences of using an unsanctioned overdose prevention site in Toronto, Ontario. Int J Drug Policy.

[38] Bourgois P (2000). Disciplining addictions: the bio-politics of methadone and heroin in the U.S.. Cult Med Psychiatry.

[39] Pearson Jeske C, O’Byrne P (2019). Perceptions and experiences of methadone maintenance treatment: a qualitative descriptive research study. J Addict Nurs.

[40] Canadian Association of People Who Use Drugs. Hear us, see us, respect us: Respecting the expertise of people who use drugs. Dartmouth, https://zenodo.org/record/5514066#.Yq4eXS8r31x (2021).

[41] Hales J, Kolla G, Man T, et al. Safer opioid supply programs (SOS): a harm reduction informed guiding document for primary care teams. 2020; 38.

[42] Ferguson M, Parmar A, Papamihali K (2022). Investigating opioid preference to inform safe supply services: a cross sectional study. Int J Drug Policy.

[43] Ranger C. Splitting & sharing in overdose prevention and supervised consumption sites: Survey results. Dartmouth, https://zenodo.org/record/4750269#.Yq4nBC8r31w (2021).

[44] Haines M, O’Byrne P (2020). Harm reduction services in Ottawa: the culture of drug use. Res Theory Nurs Pract.

[45] Bardwell G, Small W, Lavalley J (2021). “People need them or else they’re going to take fentanyl and die”: a qualitative study examining the ‘problem’ of prescription opioid diversion during an overdose epidemic. Soc Sci Med.

